# The SARS-CoV-2 spike L452R-E484Q variant in the Indian B.1.617 strain showed significant reduction in the neutralization activity of immune sera

**DOI:** 10.1093/pcmedi/pbab016

**Published:** 2021-07-30

**Authors:** Gen Li, Zhongcheng Zhou, Peng Du, Meixing Yu, Ning Li, Xinxin Xiong, Hong Huang, Zhihai Liu, Qinjin Dai, Jie Zhu, Chengbin Guo, Shanyun Wu, Daniel T Baptista-Hon, Man Miao, Lam Wai Ming, Yong Wu, Fanxin Zeng, Charlotte L Zhang, Edward D Zhang, Haifeng Song, Jianghai Liu, Johnson Yiu-Nam Lau, Andy P Xiang, Kang Zhang

**Affiliations:** Guangzhou Women and Children's Medical Center, Guangzhou Medical University, Guangzhou 510620, China; Guangzhou Women and Children's Medical Center, Guangzhou Medical University, Guangzhou 510620, China; Guangzhou Women and Children's Medical Center, Guangzhou Medical University, Guangzhou 510620, China; Guangzhou Women and Children's Medical Center, Guangzhou Medical University, Guangzhou 510620, China; Guangzhou Women and Children's Medical Center, Guangzhou Medical University, Guangzhou 510620, China; Guangzhou Women and Children's Medical Center, Guangzhou Medical University, Guangzhou 510620, China; Guangzhou Women and Children's Medical Center, Guangzhou Medical University, Guangzhou 510620, China; Guangzhou Women and Children's Medical Center, Guangzhou Medical University, Guangzhou 510620, China; Guangzhou Women and Children's Medical Center, Guangzhou Medical University, Guangzhou 510620, China; Guangzhou Women and Children's Medical Center, Guangzhou Medical University, Guangzhou 510620, China; Guangzhou Women and Children's Medical Center, Guangzhou Medical University, Guangzhou 510620, China; Guangzhou Women and Children's Medical Center, Guangzhou Medical University, Guangzhou 510620, China; University Hospital and Center for Biomedicine and Innovations, Faculty of Medicine, Macau University of Science and Technology, Macau 030027, China; University Hospital and Center for Biomedicine and Innovations, Faculty of Medicine, Macau University of Science and Technology, Macau 030027, China; University Hospital and Center for Biomedicine and Innovations, Faculty of Medicine, Macau University of Science and Technology, Macau 030027, China; Jinan University First Affiliated Hospital, Jinan University, Guangzhou 510630, China; Department of Clinical Research Center, Dazhou Central Hospital, Dazhou 635099, China; Guangzhou Women and Children's Medical Center, Guangzhou Medical University, Guangzhou 510620, China; Guangzhou Women and Children's Medical Center, Guangzhou Medical University, Guangzhou 510620, China; Department of Bioinformatics and AI, Bioland Laboratory, Guangzhou 510000, China; ABLINK Biotech Co., Chengdu 610000, China; Department of Applied Biology and Chemical Technology, Hong Kong Polytechnic University, Hung Hom, Hong Kong 610051, China; Center for Stem Cell Biology and Tissue Engineering, Key Laboratory for Stem Cells and Tissue Engineering, Ministry of Education, Sun Yat-sen University, Guangzhou 510000, China; Guangzhou Women and Children's Medical Center, Guangzhou Medical University, Guangzhou 510620, China; University Hospital and Center for Biomedicine and Innovations, Faculty of Medicine, Macau University of Science and Technology, Macau 030027, China

**Keywords:** SARS-CoV-2, COVID-19, B.1.617.1, L452R, E484Q, infectivity, immune, neutralization

## Abstract

To assess the impact of the key non-synonymous amino acid substitutions in the RBD of the spike protein of SARS-CoV-2 variant B.1.617.1 (dominant variant identified in the current India outbreak) on the infectivity and neutralization activities of the immune sera, L452R and E484Q (L452R-E484Q variant), pseudotyped virus was constructed (with the D614G background). The impact on binding with the neutralizing antibodies was also assessed with an ELISA assay. Pseudotyped virus carrying a L452R-E484Q variant showed a comparable infectivity compared with D614G. However, there was a significant reduction in the neutralization activity of the immune sera from non-human primates vaccinated with a recombinant receptor binding domain (RBD) protein, convalescent patients, and healthy vaccinees vaccinated with an mRNA vaccine. In addition, there was a reduction in binding of L452R-E484Q-D614G protein to the antibodies of the immune sera from vaccinated non-human primates. These results highlight the interplay between infectivity and other biologic factors involved in the natural evolution of SARS-CoV-2. Reduced neutralization activities against the L452R-E484Q variant will have an impact on health authority planning and implications for the vaccination strategy/new vaccine development.

## Introduction

The infection caused by the novel coronavirus SARS-CoV-2 has created a global pandemic since late 2019, and as of 15 June 2021, there were more than 175 million confirmed infections and more than 3.8 million deaths worldwide (http://covid19.who.int). Recently, a SARS-CoV-2 Variant of Interest (VOI) (https://www.who.int/en/activities/tracking-SARS-CoV-2-variants/),^1^ B.1.617.1, has emerged in India and infected millions of the population in a short period of time. Molecular characterization showed non-synonymous substitutions G142D, E154K, L452R, E484Q, D614G, P681R, and Q1071H in the spike (S) protein.^2^

In an earlier publication by Korber *et al*.,^3^ the non-synonymous substitution in amino acid position 614 of the COVID-19 spike protein from D614 to G614 led to formation of S^G614^, which is more stable than S^D614^, and potentially more efficient transmission based on pseudovirus assays.^3^, ^4^ Fortunately, both D614 and G614 were neutralized by convalescent plasma with comparable efficiencies.^3^, ^4^ At present (June 2021), D614G is more prevalent from a global perspective and associated with higher calculated non-synonymous variations of 22.2 substitutions/year vs 13.5 substitutions per year in those without D614G.^5^ Note that the B.1.617.1 strain has D614G in the S protein.

The RBD of the spike protein is the motif engaged to bind to the receptor angiotensin-converting enzyme 2 (ACE2) on host cells.^6^ Of the synonymous variations observed in the B.1.617.1 strain, L452 and E484 are located within RBD (Supplementary Fig. S1). The non-synonymous variant L452R was previously observed in other isolates including the CAL.20C (B.1.427/429) variant.^7^, ^8^ The non-synonymous variant E484K was observed in B.1.351 and P.1 variant,^9^ whereas E484Q was observed in the B.1.617.3 lineage, and in India, and composed up to 90% of the variant's sequences.^2^

Each of the L452R and E484K variants alone were shown to lead to a significant decrease in the neutralization activities of immune sera.^7^, ^8^, ^10^ We have previously reported that L452R can enhance infectivity.^8^ It remains critical for the scientific community to determine the implications of combined L452R with E484K or E484Q on the infectivity and neutralization activity of immune sera from convalescent patients. Therefore, we have constructed pseudotyped viruses with and without both L452R and E484Q in the backbone of the B.1.617 strain (with D614G) of the SARS-CoV-2 S protein to evaluate the effect of the L452R-E484Q variant on infectivity and changes in neutralization activities of immune sera from non-human primates vaccinated with a RBD protein vaccine, patients who recovered from COVID-19 infection, and vaccine recipients who received the complete two doses of Pfizer-BioNTech mRNA vaccine.

## Methods

### Construction of the COVID-19 variant S protein expression plasmids

The codon optimized full-length S protein gene was synthesized and cloned into the pCAGGS vector (Genscript, Nanjing, China). A site-directed mutagenesis approach was employed to generate D614G and L452R-E484Q-D614G mutants, with primer sequences detailed in Supplementary Table S1. The PCR Mix and recombinant enzyme used were high-fidelity DNA polymerase Mix (P525, Vazyme) and Exnase II (C214, Vazyme). All plasmid sequences were confirmed by Sanger's method.

### Human subjects

This study was approved by the Medical Ethics Committees of the Guangzhou Women and Children's Hospital and University Hospital of Macau University of Science and Technology. The patients with COVID-19 enrolled were diagnosed between January and March 2020 and were managed at the designated hospitals in Wuhan and surrounding cities in the Hubei Province,^11^, ^12^ and all COVID-19 diagnoses were confirmed by RT-PCR. The demographic and clinical information was reported previously.^11^, ^12^ Sera from vaccine recipients were obtained from volunteers with an average of 23 days after they have completed their two-dose vaccination in the University Hospital of the Macau University of Science and Technology.

### Sera from RBD-immunized non-human primates

All procedures involved in the non-human primate study were reviewed and approved by the Institutional Animal Care and Use Committee of Institute of Sun Yat-sen University. Six adult non-human primates *Cynomolgus macaques (Macaca fascicularis)* (5–9 years old) were employed for the vaccine study, which were immunized with 20 μg RBD protein with Al(OH)_3_ (n = 6). The detailed methods for vaccination, booster administration, and other experimental details were identical to those provided in our recent report.^6^

### Production and quantification of pseudotyped viruses

5 × 10^6^ 293T cells in a 100 mm dish were co-transfected with 12 μg pLOVE-luciferase-EGFP plasmid, 6 μg psPAX2 and 2 μg S or S variants plasmids using Lipofectamine 3000 (Invitrogen, L30000015) according to the manufacturer's instructions. The medium for transfected cells was replaced with 10 ml of fresh medium after 6–8 hours, and the supernatant containing SARS-CoV-2 pseudotyped viruses was harvested and filtered through a 0.45 μm filter 48 hours after transfection. RNA of 100 μl SARS-CoV-2 pseudotyped virus and the related constructed pseudotyped viruses were extracted using the MiniBEST Viral RNA/DNA Extraction Kit Ver.5.0 (TaKaRa, 9766). Reverse transcription was conducted using the HiScript® III All-in-one RT SuperMix Perfect for qPCR (Vazyme). RT-PCR was performed using a TransLv Lentivirus qPCR Titration Kit (TransGen, FV201).

### Infectivity assay

The 293T-ACE2-TMPRSS2 cells (1 × 10^4^/100 μl/well) were seeded in 96-well plates. After quantification by RT-PCR, the pseudotyped viruses were diluted to 80 000 particle number in 100 μl DMEM medium, and 100 μl of the virus suspension was added per well into the 96-well cell culture plates. After 12 hours of infection, the medium in each well was replaced with 10 ml fresh culture medium. Luciferase activity was assayed after another 48 hours of incubation in a tissue culture chamber (5% CO_2_ at 37°C). Luciferase substrate was mixed with cell lysis buffer (Promega, E6120) and was added to the plate (100 μl/well). After two minutes, 100 μl of lysate was transferred to an opaque 96-well plate and the luminescence signal was detected using TECAN Infinite P500.

### Neutralization assay

The effects of the sera and monoclonal antibodies on entry inhibition by the pseudotyped viruses were evaluated through measurement of a reduction in the luciferase gene expression. The 293T-ACE2-TMPRSS2 cells (1 × 10^4^/100 μl/well) were seeded in 96-well plates. The samples were serially diluted (50-fold as the initial dilution) for a total of eight gradients in 96-well plates. The virus solution was subsequently added to the wells. Six virus control wells (without antibody samples) and six control wells (cells without virus or antibody samples) were also included for each 96-well plate. The 96-well plates were incubated at 37°C for 1 hour. After incubating in a tissue incubator (5% CO_2_ at 37°C) for 12 hours, the medium was replaced with fresh medium, and 48 hours later, luminescence was measured as described above. The sample ED_50_ (median effective dose) was calculated using the Reed-Muench method.^13^

### ELISA

96-well ELISA plates were coated with SARS-CoV-2 Spike RBD protein (Sino Bio. 40592-V08B), RBD (E484Q) (Sino Bio. 40592-V08H81), RBD (L452R-E484Q) (Sino Bio. 40592-V08H88). Non-specific absorption of the plates was blocked with BSA and washed. Serum samples were diluted starting from 1:2000 and performed with 1:2 serial dilutions using dilution buffer. Diluted samples were added to the corresponding wells and incubated for 1 hour at 37°C, followed by washing. Antibodies were detected with Goat anti-Monkey IgG H&L (conjugated to alkaline phosphatase) where appropriate and diluted 1:1000 for a 30 minute incubation at room temperature. After washing, alkaline phosphatase yellow (pNPP) liquid substrate (Sigma, P7998-100ML) was added to each well and incubated for 15–20 minutes before the reaction was stopped using 3M NaOH. Optical density was measured at 405 nm.

### Quantification and statistical analysis

GraphPad Prism 8 was used for plotting and statistical analysis; the values were expressed as means ± SEM. A non-parametric test (Wilcoxon) was employed and a *P* value of < 0.05 was considered to be significant (*P < 0.05, **P < 0.01, ***P < 0.005, ^****^P < 0.001).

## Results

### Infectivity of the SARS-CoV-2 pseudotyped viruses with the L452R-E484Q variant

Pseudotyped virus expressing wild-type S^D614^ was used as the wild-type virus control. The D614G variant pseudotyped virus was also constructed as another control. Pseudotyped virus with the L452R/E484Q variant with the D614G background was also constructed (Supplementary Fig. S1).

**Figure 1.**
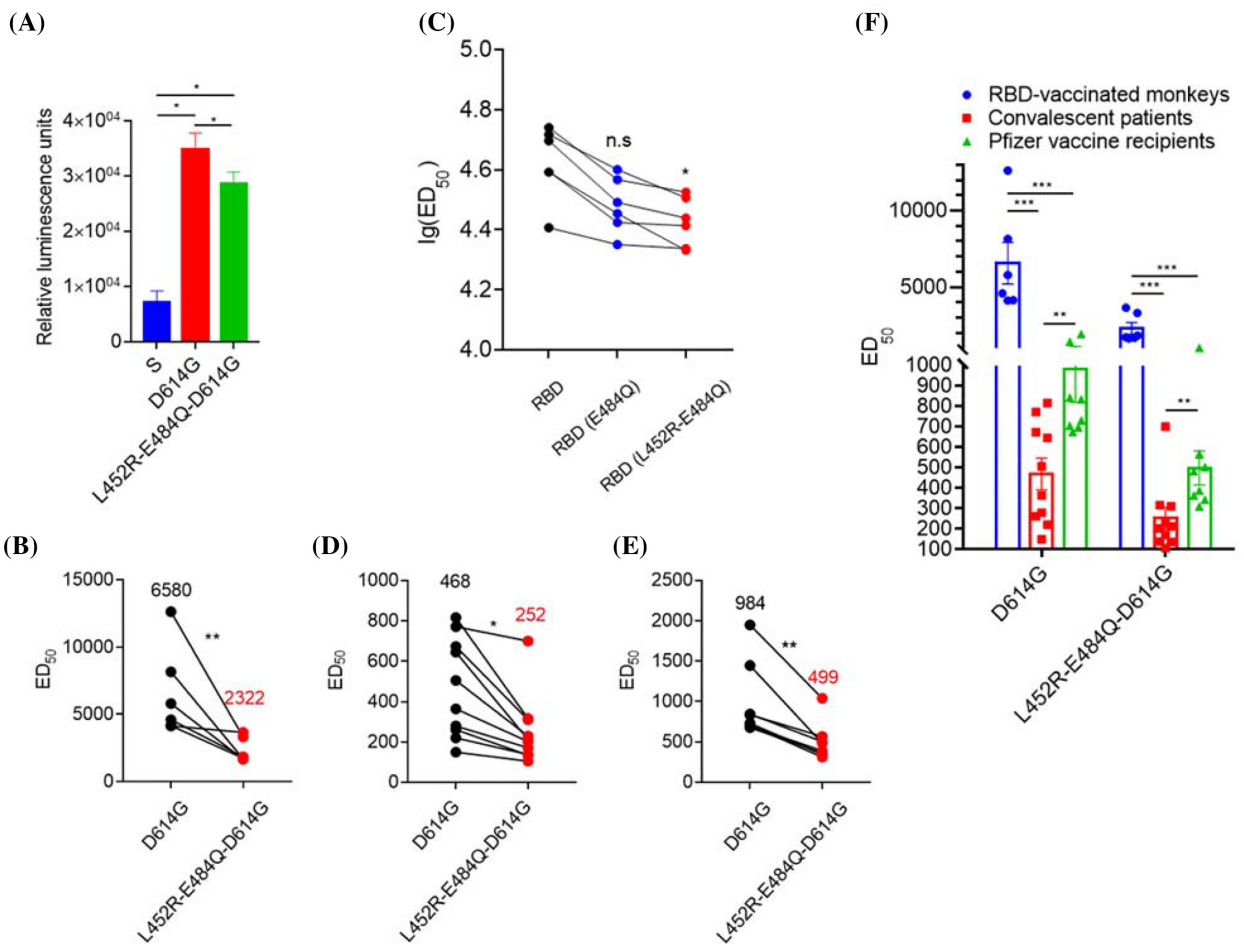
Infectivity of and effect on neutralizing activities of the immune sera for the L452R-E484Q variant with the D614G background. A. Infectivity of the pseudotyped virus. The X-axis shows different COVID-19 S variants and the Y-axis shows relative luminescence units as determined by the infectivity assay (compared with the cell culture control). B. Neutralization activities of the immune sera from non-human primates vaccinated with an RBD protein vaccine (n = 6). C. Binding affinity (ED50) of RBD, RBD (E484Q), and RBD (L452R-E484Q) with immune sera from non-human primates as determined by ELISA (n = 6). The average values of ED_50_ were labeled up to the dots. D. Neutralization activities of the immune sera from COVID-19 infection convalescent patients against D614G and L452R-E484Q (in D614G background) variants (n = 10). The average values of ED_50_ were labeled up to the dots. E. Neutralization activities of the immune sera from vaccinees (n = 8, Pfizer-BioNTech) against D614G and L452R-E484Q (in D614G background) variants. The average values of ED_50_ were labeled up to the dots. F. Summary of neutralization activities of the immune sera from the RBD-vaccinated monkeys (n = 6), COVID-19 convalescent patients (n = 10), and mRNA vaccine recipients (n = 8) against D614G and L452R-E484Q-D614G COVID-19 S variants. For Fig. 1B–F, the X-axes show different COVID-19 S variants and the Y-axes show ED_50_ or lg(ED_50_), where ED_50_ stands for 50% effective neutralization as determined by serial dilution. (*P < 0.05, **P < 0.01, ***P < 0.005, ^****^P < 0.001).

D614G had a much higher infectivity than the wild-type S^G614^ (4.9×, Fig. 1A). The addition of the L452R/E484Q variants also showed a significant increase in infectivity compared with the wild-type S protein pseudotyped virus, but slightly lower compared with the D614G variant (Fig. 1A).

### Reduced immune reactivity to the SARS-CoV-2 L452R-E484Q-D614G variant with RBD-vaccinated non-human primate sera

We then tested the impact of the variant amino acid substitutions on the neutralization activity of the immune sera obtained from non-human primates immunized with a recombinant RBD protein vaccine (conjugated to a His-tag). High anti-RBD antibody levels can be routinely obtained in these vaccinated monkeys as reflected in its ability to neutralize the infection from the pseudotyped virus.^6^ Figure 1B shows that when compared with D614G pseudovirus, L452R-E484Q-D614G pseudovirus showed marked reduced neutralization activity by the immune sera from vaccinated non-human primates, with a median of 2.8-fold.

To further characterize the role of L452R-E484Q in binding of the neutralizing antibodies to the S protein variant, the binding affinity of RBD, RBD (E484Q), and RBD (L452R-E484Q) were tested by ELISA. We have previously shown that the RBD variant L452R exhibited lower binding activity compared with the wild-type RBD.^8^ Figure 1C shows that there was a reduction of binding affinity of the immune sera to RBD (E484Q) and the covariant (L452-E484Q) showed an even lower binding affinity (n = 6).

### Neutralization activity of immune sera from convalescent patients and mRNA vaccinated subjects against the L452R-E484Q variant

The effects of L452R-E484Q variant (with D614G background) pseudotyped viruses on the viral neutralizing activities of immune sera from patients who had recovered from COVID-19 infection (n = 10) and from healthy subjects who had been vaccinated with the Pfizer mRNA vaccine (n = 8) were also evaluated. For convalescent sera, there was a significant loss of neutralization activity with the L452R-E484Q variant compared with the D614G background, with a median reduction of 1.9-fold (Fig. 1D). For sera from vaccinees, there was also a significant reduction of neutralization activity against the L452R-E484Q variant compared with the D614G background, with a median reduction of about 2.0-fold (Fig. 1E).

Figure 1F shows a summary of the neutralization activities of sera from the RBD-vaccinated monkeys, COVID-19 convalescent patients, and COVID-19 vaccine (mRNA based) recipients. Note that the mRNA showed a higher average level of neutralizing activity than the convalescent patients (all < 4–6 weeks but the two groups were not controlled for the timing of sera collection versus the viral/vaccine immune challenge) and also that the immune sera from all groups still showed neutralizing activity, even at statistically significant lower levels.

## Discussion

This study highlighted a few significant points on this important SARS-CoV-2 variant B.1.617.1., which is prevalent in the latest outbreak in India (May–June 2021). First, although we have previously shown that L452R variant can enhance viral infectivity,^8^ the L452R-E484Q variant did not cause further increase in infectivity with the background of D614G variant.^3^ Second, the L452R-E484Q variant, located on the RBD, was shown to induce a reduction in the neutralizing activity of immune sera from the non-human vaccinated primates, convalescent patients, and mRNA vaccine recipients. Third, despite the reduction of neutralizing activity of the immune sera, such activity could still be observed in the immune sera from both the convalescent patients and vaccinees.

Similar to the other three globally emerging major SARS-CoV-2 variants of concern (VOC) (B.1.1.7, B.1.351, and P.1) reported recently,^14–16^ B.1.617.1 variant, the major VOC that contributed to the latest outbreak in India, was found to have a number of non-synonymous amino acid substitutions in the spike protein, with a couple of these within the RBD. L452R was previously found to have an impact on enhancing viral infectivity; however, addition of the amino acid substitution E484Q did not seem to further enhance this (in fact, the infectivity of the L452R reduced slightly). Assuming the theory of survival fitness for the viral evolution, the evolution of E484Q in the latest outbreak suggests that there are other viral factors (replication, structural fitness) and virus-host interactions that provided the survival advantage. One of the possible factors, in terms of reduction of the host immune sera neutralization activity, was observed with this L452R-E484Q variant in our study. Another important mutation in B.1.617.1, P681R, may facilitate furin-mediated spike cleavage, and enhance and accelerate cell-cell fusion.^17^ The function of the S2 mutation Q1071H and up to three NTD substitutions (T95I, G142D and E154K) is so far unclear. Very recently, Liu *et al*. reported that the neutralizing titer of Pfizer-BNT162b2 vaccine-elicited sera against B.1.617.1-spike was reduced compared with D614G,^18^ and also there was a report of reduced neutralization of B.1.617.1 by vaccine and convalescent serum,^19^ both findings in accordance with our observations. We and others have previously shown that L452R could reduce the neutralization activities of immune sera.^7^, ^8^ In this report, we are also adding E484Q as part of the “Immune escape” variants, and in the case of B.1.617.1, the immune escape variant. The more uncommon variant, E484K, was also recently reported to have a similar profile.^16^, ^17^ One can certainly not exclude the possibility that there are other viral and host factors involved in the viral evolution leading to the B.1.617.1. strain. The observation of this immune escape in the context of a major outbreak in India suggests the possibility that a tilt of the viral-host interaction may potentially cause a major outbreak, hence the viral, host, and viral-host interaction factors should be closely and rapidly monitored, so that the health authorities can adjust to this pandemic in a speedy fashion.

It is important to note that based on our assay methods, the immune escape of the L452R-E484Q was not complete and that all the immune sera still showed neutralization activities. On one hand, one can argue that the current vaccines are likely protecting against the Indian variant. On the other hand, it is possible that the protection offered by the neutralizing antibodies in convalescent patients and vaccinees against the B.1.617.1 strain may be shorter in duration compared with the wild-type SARS-CoV-2. This will have public health impact with regards to the duration of the protection in convalescent patient and vaccinees as these people may also need to protect themselves from being infected and serve as an infection sources in an outbreak. Certainly, these findings will have implications on the need and timing for vaccine boosters and the needs for additional vaccines targeting these variants.

One potential limitation is that this study was conducted using pseudotyped viruses and the binding assay was conducted *in vitro* based on ELISA. However, these systems have been previously shown to correlate well with the *in vivo* situation and the speedy generation of data can certainly help the health authorities and also vaccine developers to plan and prepare ahead of the schedule.

## Supplementary Material

pbab016_Supplemental_File
