# Supplementary material for: The SARS-CoV-2 spike L452R-E484Q variant in the Indian B.1.617 strain showed significant reduction in the neutralization activity of immune sera
Source: Precis Clin Med. 2021 Jul 30;4(3):149–54. doi: 10.1093/pcmedi/pbab016 (PMC8385834; doi:10.1093/pcmedi/pbab016)
Supplement: pbab016_Supplemental_File [file pbab016_supplemental_file.docx]

**Supplementary Materials**

**Figure S1. A graphical presentation of the L452R and E484Q sites based on the solved complex structure of SARS-CoV-2 RBD bound to ACE2 (PDB code: 6LZG)**


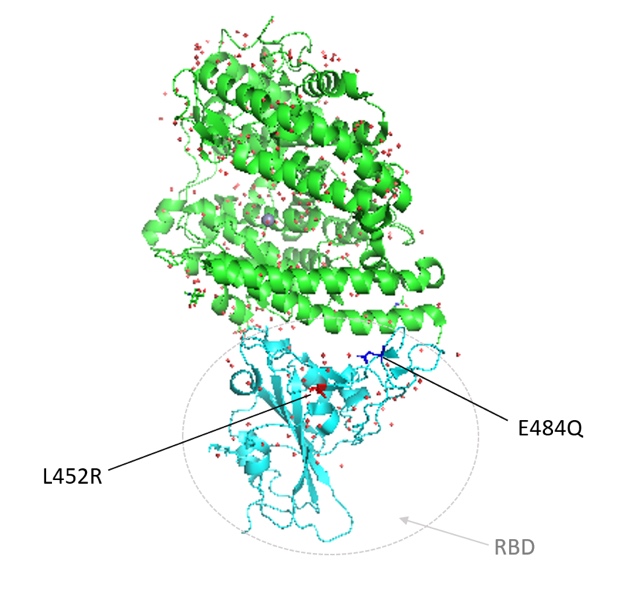


ACE2 labeled as Green, RBD labeled as Cyan, and L452R and E484Q sites were labeled as Red and Blue, respectively.

**Supplementary Table 1. Primers used for the generation of the mutant S variants plasmids.**

| S-E484Q-F | TGCAATGGCGTGCAGGGCTTTAACTGTTATTTCCCAC |
| --- | --- |
| S-E484Q-R | AGTTAAAGCCCTGCACGCCATTGCAAGGGGTGCTGCC |
| S-L452R-F | ACTACAATTATCGGTACCGGCTGTTTAGAAAGAGCAA |
| S-L452R-R | AACAGCCGGTACCGATAATTGTAGTTGCCGCCCACTT |
| S-D614G-F | TGCTGTATCAGGGCGTGAATTGTACCGAGGTGCCCGT |
| S-D614G-F | GTACAATTCACGCCCTGATACAGCACGGCCACCTGGT |
